# A Pop from a Shock: A Case Report of an Unusual Cause of Achilles Tendon Rupture

**DOI:** 10.5811/cpcem.2022.2.55090

**Published:** 2022-04-06

**Authors:** Chia-Yuan Michael Lee, Mark A. Newberry

**Affiliations:** Mount Sinai Medical Center, Department of Emergency Medicine, Miami Beach, Florida

**Keywords:** case report, Achilles tendon injury, electric shock injuries, point-of-care ultrasound

## Abstract

**Introduction:**

Achilles tendon ruptures often occur during physical activity where the tendon is frequently stressed. Although rare, rupture can also result from electric shock.

**Case Report:**

We present the case of a 63-year-old female who presented with pain in the lower leg after enduring an electric shock. She was diagnosed with a ruptured Achilles tendon based on physical exam and ultrasound.

**Conclusion:**

This case highlights an uncommon mechanism for a relatively common injury. Because Achilles tendon ruptures are frequently misdiagnosed, clinicians need to be aware of unusual causes and use tools at their disposal to ensure timely and accurate diagnosis.

## INTRODUCTION

Despite being the thickest and strongest tendon of the human body, the Achilles tendon (AT) is the one that most often completely ruptures.^1^,^2^ Because it is at an increased risk of rupture during excessive eccentric muscle contraction or explosive plyometric movements, most AT ruptures occur during physical activity.^3^ Here, we present a case of AT rupture after an uncontrolled and unexpected muscle contraction triggered by the unintentional exposure to a low-voltage household current.

## CASE REPORT

A 63-year-old female presented to the emergency department (ED) with pain just posterior to the left medial malleolus after experiencing an electric shock. She had stepped on an exposed wire on her way to bed after a shower and felt a shock followed immediately by a left calf spasm. She denied falls, syncope, or additional blunt trauma to her leg. Initially, she thought it was a minor cramp that would resolve. Although she was able to ambulate, persistent discomfort and new-onset bruising behind the malleoli prompted her to go to the ED the next day.

On evaluation, her vitals were unremarkable. There was decreased plantarflexion both actively and when her calf was squeezed. An area of ecchymosis was present above her calcaneus and posterior to her medial malleolus. There were no burns or wounds noted at the electrocution site. Her labs, including a complete blood cell count, basic metabolic panel, and creatine phosphokinase were unremarkable. Her electrocardiogram revealed a normal sinus rhythm.

**Image 1:**
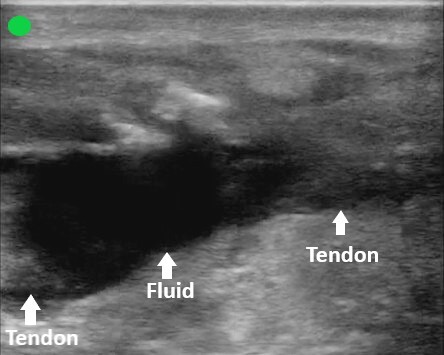
Sagittal view of the proximal Achilles tendon. Point-of-care ultrasound image obtained with a high-frequency linear probe with the probe marker directed cephalad. A large anechoic fluid collection is present between two distinct ends of the ruptured tendon.

**Image 2:**
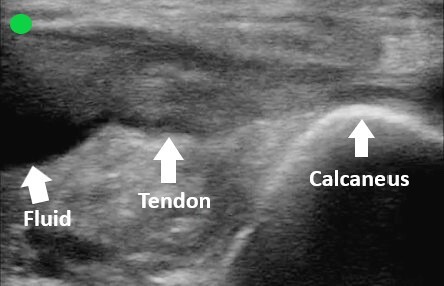
Sagittal view of the distal Achilles tendon. Point-of-care ultrasound image obtained with a high-frequency linear probe with the probe marker directed cephalad. An anechoic fluid collection is present just proximal to where the Achilles tendon attaches to the calcaneus.

**Image 3:**
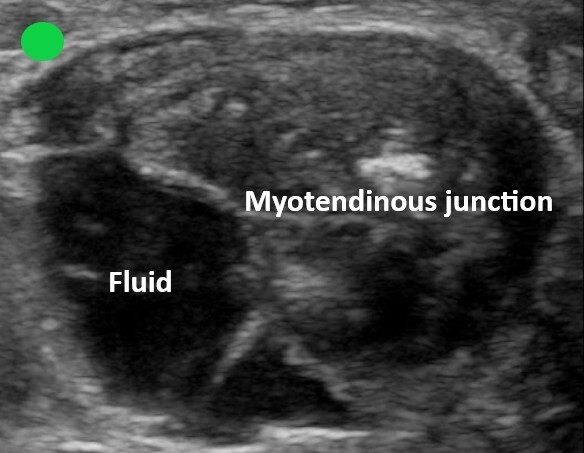
Transverse view of the Achilles myotendinous junction. Point-of-care ultrasound image obtained with a high-frequency linear probe with the probe marker directed to the left of a prone patient. A large anechoic fluid collection is present among the fibers of the myotendinous junction.

The overall clinical picture was suspicious for AT injury, prompting the use of point-of-care ultrasound to evaluate the integrity of the tendon in both the sagittal and transverse planes. The images obtained supported the diagnosis of a complete AT rupture (Image 1, 2, and 3).

The patient was placed in a posterior short leg splint with her foot in plantarflexion and discharged with crutches. She was instructed to follow up with an orthopedic surgeon.

## DISCUSSION

Achilles tendon ruptures are a relatively common injury with an estimated incidence between seven and 40 per 100,000 people per year.^3^ Over the past few decades, the incidence of ruptures has risen significantly, likely due to increased participation in physical activity.^3^,^4^,^5^ Multiple studies have demonstrated that most AT ruptures occur during sports.^2^,^3^,^4^,^6^,^7^ Approximately 80% of AT ruptures occur two to six centimeters above its insertion point at the calcaneus as this region is vulnerable to injury due to its relatively poor blood supply.^2^,^4^,^6^ The injury predominantly occurs in males with a male-to-female ratio ranging from 2:1 to 12:1.^1^,^4^ White-collar professionals who only intermittently engage in athletics comprise a significant portion of these patients.^1^,^6^

The most frequent mechanism of injury is pushing off the forefoot while extending the knee, a movement commonly performed in sports such as basketball, soccer, volleyball, and gymnastics.^1^ Other common mechanisms of injury include abrupt unexpected dorsiflexion (eg, stumbling into a hole or slipping down steps) and extreme dorsiflexion of a foot in plantarflexion (eg, falling from height).^1^

**CPC-EM Capsule**

What do we already know about this clinical entity?

*Achilles tendon ruptures frequently occur during physical activity*.

What makes this presentation of disease reportable?

*Achilles tendon rupture resulting from an involuntary muscle contraction secondary to an electric shock is an uncommon and unusual mechanism of injury*.

What is the major learning point?

*Achilles tendon ruptures are frequently misdiagnosed. Simple physical exam maneuvers and ultrasound can help make the diagnosis*.

How might this improve emergency medicine practice?

*Familiarity with uncommon causes of Achilles tendon rupture and competency with point-of-care ultrasound may expedite time to diagnosis and prevent delay in proper management*.

While the exact etiology for tendon rupture is unclear, there are intrinsic and extrinsic factors that can predispose an individual to tendinopathy and injury.^4^,^7^ Intrinsic factors include anatomical malalignments, biomechanics, weight, age, or gender.^7^ Certain conditions such as autoimmune and inflammatory disease, renal insufficiency, and gout may also increase risk of rupture.^7^,^8^ Extrinsic factors consist of training errors, environment, and medications such as steroids.^4^,^7^ The patient in this case had no known predisposing factors other than her age.

In a noteworthy scenario, our patient suffered a rupture from an electric shock. To the best of our knowledge, there has only been one other case report of AT rupture from an electric shock. In that case, a woman experienced a shock while blow drying her hair, causing her to jerk her leg uncontrollably and rupture her tendon.^9^

Approximately 10,000 patients present annually to the ED for evaluation after experiencing an electric shock, which typically occurs at home or work.^10^ Electric shock injuries (ESI) can result from thermal energy due to heat, electrical damage from the current, or mechanical damage from trauma or forced contractions.^10^ The extent of injury depends on factors including resistance, voltage, current type, and duration of contact.^10^–^12^ Although ESI most commonly present as skin burns, it is common for ESI victims to also experience bone, muscle, or tendon injury.^10^ High-voltage currents will typically cause burns while low-voltage currents are more likely to cause muscle tetany.^11^ Most household electrical sources consist of alternating current, which is more damaging than direct current given equal voltages.^11^ Furthermore, household currents are typically low frequency, which tend to cause muscle contractions.^11^ At times, the contractions may even be strong enough to cause fractures and dislocations.^10^–^12^

The classic presentation of an AT rupture is sudden pain in the leg that may be associated with a pop or snap. Patients may report feeling as if they were struck in the calf or heel. They may struggle with walking and pushing off the affected leg. Approximately 25% of AT ruptures are initially misdiagnosed, frequently as ankle sprains.^1^,^5^,^6^ Unexpected mechanisms, lack of pain, and ability to ambulate or plantarflex may confuse clinicians and lead to error.^5^,^6^ For the emergency clinician, it is of utmost importance to avoid missing the diagnosis. Failure to diagnose or delay in treatment can result in chronic issues such as pain, ambulatory dysfunction, loss of strength, and difficulty in returning to baseline activities.^5^ Thus, the emergency clinician should be familiar with physical exam findings and imaging modalities that can assist in identifying an AT rupture.

Clinical diagnostic tests that can be used to evaluate for AT rupture include palpating for a defect along the tendon, the calf squeeze test, Matles test, Copeland test, and O’Brien test. The calf squeeze test and Matles test have the highest sensitivity and, unlike the Copeland and O’Brien tests, do not require additional equipment.^13^ To perform the calf-squeeze test, the examiner should squeeze the patient’s calf to induce plantarflexion of the foot. If the AT is ruptured, there should be no or little plantarflexion induced.^13^ To perform the Matles test, the patient should lie prone and flex both knees to 90 degrees. The examiner should then evaluate the position of the feet. If the AT is intact, the foot should be in slight plantarflexion, and if the AT is ruptured, the foot should be in a neutral position or in dorsiflexion.^13^

If clinical testing is ambiguous or limited due to pain or body habitus, further imaging, such as clinical ultrasound (CUS) and magnetic resonance imaging (MRI), can be considered. Although MRI is the gold standard for imaging, it is more expensive and time consuming than CUS and less likely to be available.^5^,^8^ Clinical ultrasound provides real-time analysis, allows for efficient dynamic examination of the tendon, and has been shown to have high diagnostic capability with some studies reporting a sensitivity of up to 100%.^8^,^14^ Musculoskeletal ultrasound is routinely used by emergency physicians and taught in emergency medicine residency programs; it is included as a core application for emergency physicians in the most recent emergency, point-of-care and clinical ultrasound guidelines.^15^

To perform ultrasound evaluation of the AT, the patient should lie prone. The fibers of the tendon should be evaluated in both a sagittal and transverse plane. For assessment in the sagittal plane, the probe should be placed directly on top of and in line with the tendon. For assessment in the transverse plane, the probe should be placed perpendicular to the tendon. Using a high-frequency linear probe, the examiner should start scanning at the calcaneal tuberosity where the AT inserts and then slide the probe proximally to the myotendinous junction. A normal AT will have fibers that are linear, regular, and without interruption. A ruptured tendon may have fibers with thick irregular edges, interruptions along the course of the tendon, or anechoic areas that may represent hematoma.

Once the diagnosis is made, the emergency clinician should splint the patient’s affected extremity in the equinus position (plantarflexion), provide crutches and analgesics, and refer the patient to an orthopedic surgeon for follow-up ideally within two days.^6^ Definitive treatment of the injury centers on restoring the tendon to its normal length and tension and rehabilitating calf muscle strength and function.^6^ Currently, there is no consensus on whether a conservative or operative approach is the optimal treatment for acute AT ruptures.^1^,^2^,^8^ Ultimately, patient-specific factors and shared decision-making with an orthopedic surgeon should determine the definitive treatment plan.

## CONCLUSION

Although Achilles tendon ruptures are common, not all result from obvious mechanisms. Uncommon and atypical mechanisms, such as an electric shock, can also lead to tendon injury. Emergency clinicians need to remain astute, so that they do not miss the diagnosis and delay proper treatment. If there is doubt regarding the diagnosis, ultrasound can help confirm the presence of an AT rupture.
